# Discontinuation of preventive antiepileptic drugs in patients with intracerebral hemorrhage

**DOI:** 10.1186/s12883-021-02177-w

**Published:** 2021-04-07

**Authors:** Yi-Sin Wong, Chi-Shun Wu, Cheung-Ter Ong

**Affiliations:** 1 Department of Family Medicine, Ditmanson Medical Foundation Chia-Yi Christian Hospital, Chia-Yi, Taiwan; 2 Department of Nursing, Min-Hwei Junior College of Health Care Management, Tainan, Taiwan; 3 Department of Neurology, Ditmanson Medical Foundation Chia-Yi Christian Hospital, 539 Chung-Shao Road, Chia-Yi, Taiwan

**Keywords:** Intracerebral hemorrhage, Stroke, Seizure, Anticonvulsant, Valproic acid, Prophylaxis

## Abstract

**Background:**

The risk factors for seizures in patients with intracerebral hemorrhage (ICH) stroke and the effect of seizure prevention by anticonvulsant are not well understood. Limited studies have investigated the risk of seizure after discontinuing antiepileptic drugs in patients with ICH. This study aimed to investigate the role of valproic acid (VA) for seizure prevention and to access the risk of seizure after anticonvulsant withdrawal in patients with spontaneous ICH.

**Methods:**

Between 2013 and 2015, 177 patients with ICH were enrolled in this 3-year retrospective study. Seizures were classified as early seizure (first seizure within 1 week of ICH), delayed seizure (first seizure after 1 week), and late seizure (any seizure after 1 week). Binary logistic regression was used to evaluate the relationship between baseline clinical factors and late seizures between study periods. VA was prescribed or discontinued based on the decision of the physician in charge.

**Results:**

Seizures occurred in 24 patients, including early seizure in 6.78% (12/177) of the patients, delayed seizure in 7.27% (12/165) of the patients without early seizure, and late seizure in 9.60% (17/177) of the patients. Most seizures occurred within the first year. Binary logistic regression analysis showed ICH with cortex involvement as the independent risk factor for seizures. VA did not decrease the risk of seizures. Patients with ICH with cortical involvement using anticonvulsants for longer than 3 months did not have a decreased risk of seizures (odds ratio 1.86, 95% CI: 0.43–8.05).

**Conclusions:**

Spontaneous ICH with cortex involvement is the risk factor for seizure. Most seizures occurred within 1 year after stroke onset over a 3-year follow up. Discontinuation of antiepileptic drug within 3 months in patients does not increase the risk of seizure.

**Supplementary Information:**

The online version contains supplementary material available at 10.1186/s12883-021-02177-w.

## Background

Stroke is one of the most common causes of epilepsy. In old patients, more than 50% of the cases of seizure are related to stroke [1, 2]. The frequency of seizures after stroke was found to be approximately 4–10% in patients with ischemic stroke and 4–27% in patients with hemorrhagic stroke [3–6]. The seizures after stroke include acute symptomatic seizure (early) attack within 1 week after stroke onset and unprovoked (late) seizure onset after 1 week of stroke [5, 7, 8].

In patients with early seizure, approximately 50% seizures were found to occur at the onset of intracerebral hemorrhage (ICH) [5]. A previous study reported that patients with stroke who have experienced early seizure have a higher risk of developing late seizure than those who have not [9]. A study by Biffi et al., including 872 patients, found that after 3.9 years of follow up, approximately 50% (42/86) of the patients with early seizure experienced recurrent seizure, and 4.24% (37/872) experienced late seizure [10].

The effect of early seizure is controversial. A study by Hert et al. reported that in patients with ICH, early seizure does not influence the patient’s 6-month outcome [5, 11, 12]. However, another study showed the association between early seizure and poor outcome in a patient with ICH [13]. The factors that may increase the risk of seizure in patients with ICH include cortical involvement, intraparenchymal hemorrhage with midline shift, patients with non-neurologic infection, and hemorrhage volume [6, 14–16].

Although the guidelines for the management of ICH do not recommend the use of prophylactic anticonvulsant treatment for patients without seizures [17], prophylactic anticonvulsant treatment in patients with ICH is common. A previous study found that prophylactic anticonvulsant for seizure in patients with ICH can reduce early seizure and improve neurological outcome [18, 19].

However, some studies found that prophylactic antiepileptic agents do not reduce the occurrence of seizure. A study by Naidech et al. found that prophylactic levetiracetam in patients with ICH does not affect seizure and functional outcome but has worse cognitive function and health-related quality of life [20].

The duration for which prophylactic anticonvulsants should be used after ICH is controversial. At present, most of the existing studies have investigated the effect of prophylactic anticonvulsant on patients with ICH, who use phenytoin or levetiracetam. Few studies have investigated the effect of VA. However, there are no studies investigating the duration of prophylactic anticonvulsant in patients with ICH have been reported. Hence, we performed a study investigating the prophylactic effect of valproic acid (VA) on seizure in patients with ICH.

The aim of the study was to investigate the incidence and associated factors of early and late seizure in patients with ICH. We also investigated whether the discontinuation of the prophylactic anticonvulsant increased the risk of late seizure in patients with ICH.

## Methods

Between Jan 1, 2013 and Dec 31, 2015, 287 patients with intracerebral hemorrhage stroke were admitted to Chia Yi Christian hospital. The hospital is a 1000-bed teaching hospital in central Taiwan. This study was a retrospective study; we reviewed the patients’ medical records, including demographic data, vascular risk factors, and the process of care from stroke onset to 3 years after the stroke. Brain computed tomography (CT) was performed based on the electrical medical records.

All consecutive patients with acute neurological symptoms arrived at the emergency department and underwent brain CT, and patients with acute hemorrhagic stroke were included in the study. Patients with ICH due to trauma, tissue plasminogen activator-related hemorrhage, arteriovenous malformation rupture, subarachnoid hemorrhage (SAH), cerebellar hemorrhage, and brain stem hemorrhage were excluded from the study. All patients were evaluated by a neurosurgeon, and seizures were classified according to the criteria of an international league against epilepsy [21, 22]. Only motor onset focal (with and without impaired awareness) and generalized seizures were included in the study. The definition of seizure was according to that used in previous studies.

Early seizure (ES) was defined as the first seizure occurring within 7 days after stroke. The first seizure occurring beyond 1 week after stroke was defined as delayed seizure (DS). Late seizure was defined as seizure that occurred after 1 week of stroke onset, including patients who had experienced ES and those who had not [10]. The end point of the study was 3 years after stroke onset. If patients had no seizure till 3 years after stroke onset, they were considered to have no seizure. In the emergency department and ward, the patients or their families were inquired about seizures and the onset of stroke.

### Demographic characteristics and medical history

During hospitalization, the following information was collected: 1. Age and gender; 2. Consciousness level (Glasgow coma scale) at admission; 3. Risk factors of stroke; 4. Previous stroke history (infarct, hemorrhage, and undetermined); 5. Previous therapy before stroke. All of the patients were treated for blood pressure control, fluid and nutrition supply, airway management, and surgery according to the guidelines of the American Heart Association [23]. If seizures developed, 400 mg VA was administered twice daily, or 3 times a day. The decision of administering anticonvulsant to the patients without seizure was made by the physician in charge. When anticonvulsant was used for patients without seizure, VA was administered at 500 mg every day, 400 mg twice daily or 400 mg three times a day. The timing of discontinuation of the anticonvulsant was decided by the physician.

### Radiology assessment

Brain CT was performed soon after the patients arrived at the emergency department. Follow-up brain CT was performed if necessary, including neurological deficit worsening, seizure attack, consciousness level worsening, or if the patient received surgery. Brain CT images were reviewed in digital image by a neuroradiologist who was blinded to the patients. The hemorrhage volume for ICH was determined according to A × B × C/2 method. A represents the longitudinal diameter, B represents the diameter perpendicular to A, and C represents the number of 10-mm images containing hematoma [6, 24]. CT image showing a lobar hemorrhage and deep hemorrhage extending to the cortex indicated cortex involvement. Hematoma volume ≥ 30 cm^3^ (CC) was considered as moderate-to-severe and severe stroke [25]. The study protocol was approved by the Chia Yi Christian hospital’s Institutional Review Committee on human research (IRB2020131).

### Statistical analysis

The risk factors for seizure including sex, risk factors of vascular disease and Clinical manifestation were analyzed with the Chi-square or Fisher exact test. Patient age, and coma scale were analyzed using independent t-test. Binary Logistic regression was used to evaluate the relationship between baseline clinical factors and late seizure occurrence during study period. All statistical analyses were conducted using commercially available software, version 21 of the SPSS system for Windows (version 21.0. IBM Corporation. Somers, NY, USA). Two-sided *P* values of < 0.05 were considered statistically significant.

## Results

**Fig. 1:**
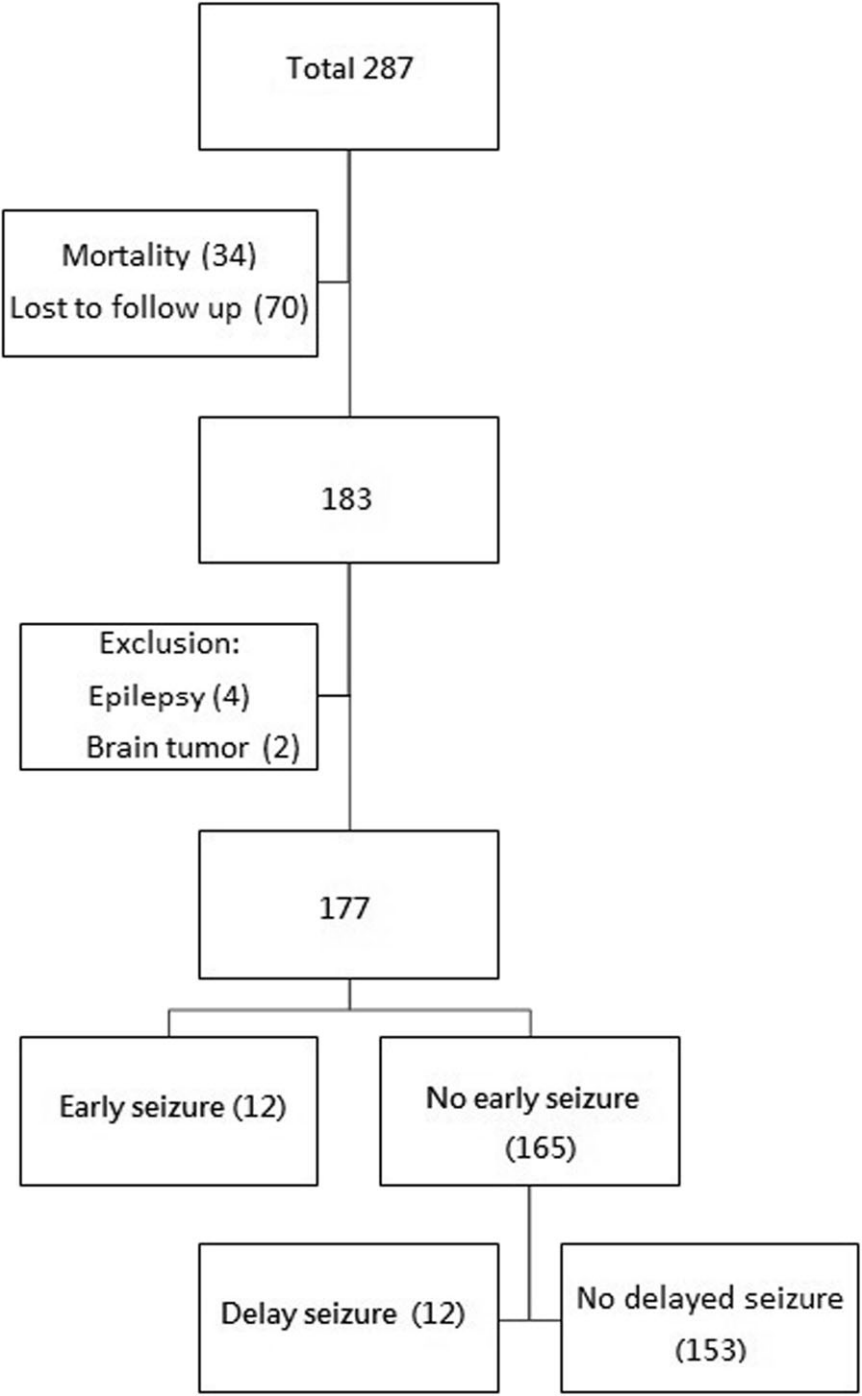
Flow chart of patient enrollment

**Fig. 2:**
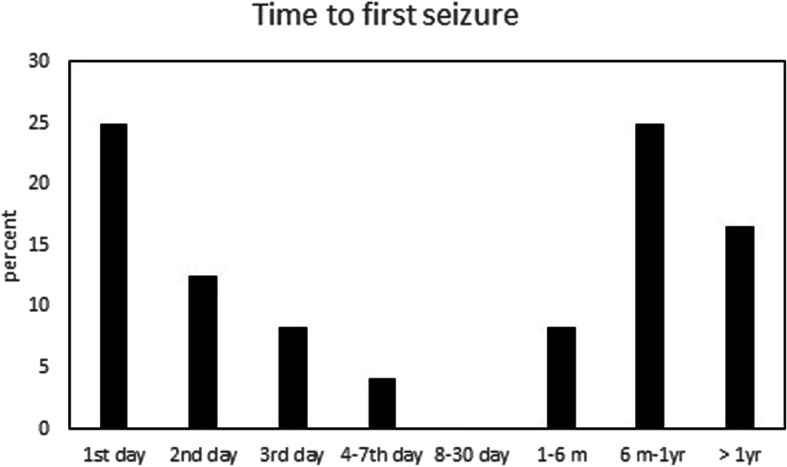
Delay of occurrence of the first seizure (*n* = 24)

Between Jan 1, 2013, and Dec 31, 2015, total 287 patients diagnosed with ICH were admitted to our hospital. After excluding the patients who died within 2 years, were lost to follow-up, or had epilepsy and brain tumor, 177 patients were included in the analysis. The flow chart of patient enrollment and exclusion is shown in Fig. 1. The characteristics of the patients with ICH are shown in Table 1. Of the 177 patients, 24 patients had seizures within 3 years after stroke onset, 12 had seizure within 1 week, 12 had the first seizure after 1 week of stroke onset, and 153 did not experience any seizures. The timing of seizure attack is shown in Fig. 2. Of the 12 patients who had ES, 50% (6/12) of the seizures occurred within 24 h after stroke onset. In the 12 patients who had DS, 66.7% (8/12) of the seizures occurred within 1 year after stroke onset.

**Table 1:** Characteristics of patients with seizures with intracerebral hemorrhage

	Total (177)	ES (*n* = 12)	P	DS (*n* = 12)	*p*
Sex					
Men	119	10		9	
Women	58	2	0.51	3	0.75
Age	63.7 ± 13.6	66.3 ± 13.5	0.51	58.6 ± 17.9	0.17
Diabetes					
No	131	9		9	
Yes	46	3	1	3	1
Atrial fibrillation					
No	167	12		11	
Yes	10	0	1	1	0.51
Hypertension					
No	20	3		4	
Yes	157	9	0.14	8	0.3
Stroke history					
No	137	9		8	
Infarct	19	2	0.75	1	0.34
Hemorrhage	21	1		3	
Volume					
< 30 CC	154	7		9	
≥ 30 CC	23	5	0.01	3	0.19
Cortex					
No	123	3		3	
Yes	54	9	0.001	9	0.001
Operation					
No	128	5		6	
Yes	49	7	0.07	6	0.09
GCS	11.9 ± 3.9	10.6 ± 3.6	0.03	12.9 ± 3.2	0.81

*ES* early seizure, *DS* delayed seizure, *GCS* Glasgow coma scale

Late seizure was found in 17 patients, including 5 recurrent seizures and 12 delayed seizures. Complete blood count and biochemical examination was performed for all of the 17 patients and we ruled out the possibility of seizures related to electrolyte imbalance, infection, alcohol withdrawal or other metabolic factors. Follow-up brain CT was performed for 14 patients and Magnetic Resonance Imaging (MRI) was performed for 1 patient to rule out the possibility of late seizure related to recurrent stroke. Two patients did not undergo follow-up brain CT because no new neurological sign was observed. In the 17 patients with late seizure, no evidence of occurrence of seizure related to recurrent stroke was found.

### Factors affecting the use of anticonvulsants

In the present study, 64 patients were not prescribed anticonvulsants, whereas 113 patients were prescribed anticonvulsants. The factors affecting the prescription of anticonvulsants were hematoma volume (*p* < 0.01), craniotomy (*p* < 0.01) and cortical involvement (*P* = 0.004). Two of the 64 patients (3.1%) who did not use anticonvulsants and 15 of the 113 patients (13.3%) received preventive anticonvulsants had late seizures (Table S1).

### Factors affecting seizure attack

Age, sex, Glasgow coma scale, diabetes mellitus, hypertension, atrial fibrillation, stroke history, stroke severity, and operation did not affect the risk of ES. Cortex involvement significantly increases the risk of ES. Hematoma ≥30 CC increased risk of early seizures (*P* = 0.01) but not of delayed seizures (*p* = 0.19) (Table 1). Of the 12 patients with ES, no recurrent seizures occurred in those 3 patients who had hemorrhages with no cortex involvement. Five of the 9 (55.5%) patients who had hemorrhage with cortex involvement had recurrent seizures (Table 2).

**Table 2:** Factors affecting recurrent seizures in patients with intracerebral hemorrhage with early seizures

	No re-seizure	Re-seizure	*p*	OR	95% CI
Anticonvulsant					
≤ 3 months	3	1	0.57	1	0.21–42.6
> 3 months	4	4		4	
Volumes					
< 30 CC	4	3		1	
≥ 30 CC	3	2	1	0.88	0.08–9.16
Cortex					
No	3	0			
Yes	4	5	0.2		
Operation					
No	3	2		1	
Yes	4	3	0.9	0.88	0.08–9.16
Hypertension					
No	1	2		0.3	
Yes	6	3	0.52	1	0.01–3.99

*OR* odds ratio, *CI* confidence interval, *Re-seizure* recurrent seizure

Sex, age, Glasgow coma scale, diabetes mellitus, hypertension, atrial fibrillation, stroke history, and operation were not significantly different between the patients with DS and those without seizure. In the patients with hemorrhage with cortex involvement, there was a significant increase in the risk of DS (*p* = 0.006). Of the 123 patients without cortex involvement, 2.43% (3/123) patients had DS, and 16.7% (9/54) of the patients with hemorrhage with cortex involvement had DS (Table 1). Under univariate analysis, cortex involvement was found to significantly increase the risk of DS with an odds ratio of 7.7 (95% CI 1.56–38.5) (Table 3).

**Table 3:** Factors affecting delayed seizures in patients with intracerebral hemorrhage who received antiepileptic medication (*n* = 113)

	No seizures (103)	Delayed seizures (10)	*P*	OR	95% CI
Sex					
Women	33	3		1.1	0.26–4.52
Men	70	7	0.89	1	
Age	63.1 ± 12.2	62.2 ± 17.5	0.81		
GCS	11.8 ± 3.4	13.5 ± 2.2	0.14		
Diabetes mellitus					
No	69	9		0.22	
yes	34	1	0.17	1	0.02–1.85
Af					
No	99	9			
Yes	4	1	0.37	2.75	0.27–27.29
Hypertension				1	
No	8	4		1	
Yes	95	6	0.01	0.12	0.03–0.54
Stroke history					
No	85	6			
Infarct	8	1			
Hemorrhage	10	3	0.14		
Anticonvulsant					
≤ 3 month	87	3		12.6	2.96–5.42
> 3 month	16	7	0.01	1	
Cortex					
No	68	2		7.7	
Yes	35	8	0.006	1	1.56–38.5
Operation					
No	62	5		0.66	
Yes	41	5	0.53	1	0.18–2.43

*GCS* Glasgow coma scale, *Af* atrial fibrillation, *OR* odds ratio, *CI* confidence interval

The factors affecting late seizure are the same as those affecting early and delayed seizures; the binary logistic regression analysis showed that cortex involvement increases the risk of late seizure with and odds ratio of 22.9 (95% CI, 3.89–135.4). Patients who used prophylactic anticonvulsant for > 3 months after stroke did not show a decrease in the risk of late seizures (Table 4).

**Table 4:** Factors affecting late seizures in patients with intracerebral hemorrhage and who received anticonvulsants (Binary logistic regression)

	No LS	LS	*p*	OR	95% CI
Stroke history					
No	81	10		1	
Ischemic	8	1	0.80	0.73	0.06–8.64
Hemorrhage	9	4	0.05	5.15	0.95–27.8
Sex					
Women	32	4		3.24	0.65–16.15
Men	66	11	0.15	1	
Atrial fibrillation					
Yes	4	1		0.72	0.06–8.23
No	94	14	0.79	1	
Volume					
≥ 30 CC	17	5		0.35	0.06–2.11
< 30 CC	81	10	0.25	1	
Operation					
Yes	38	8		2.05	0.41–10.17
No	60	7	0.37	1	
Cortex					
Yes	30	13		22.9	3.89–135.4
No	68	2	0.001	1	
Anticonvulsants					
> 3 months	50	11		2.05	0.50–8.39
≤ 3 months	48	4	0.31	1	

*LS* late seizure, *OR* odds ratio, *CI* confidence interval

### Effect of discontinuation of prophylactic anticonvulsant on late seizure

Among the 49 patients who received hematoma evacuation and used prophylactic anticonvulsants, 23.3% (6/26) of patients who were administrated VA for > 3 months had late seizures. Three of the 23 (13.0%) patients who discontinued VA within 3 months after stroke onset had late seizures. The risk of seizure was not significantly different between the patients who discontinued anticonvulsant within 3 months and those who used VA for > 3 months (*p* = 0.47, 95% CI: 0.43–9.12). Among patients with hematoma volume ≥ 30 CC, 20% (3/15) using VA for > 3 months had late seizure, and 25% (2/8) who discontinued VA within 3 months after stroke onset had late seizure. The risk of seizure was not significantly different among the patients who did not use VA or discontinued VA within 3 months and those who used VA for > 3 months (*p* = 0.75, 95% CI: 0.09–5.76). Among patients with cortical involvement, 15.4% (4/26) of patients who used VA for > 3 months had late seizures, and 35.7% (10/28) who discontinued VA within 3 months after stroke onset had late seizures. The risk of late seizure was not significantly different in the patients who discontinued VA within 3 months and the patients who used VA for > 3 months (OR = 3.05, 95% CI: 0.81–11.3) (Table 5).

**Table 5:** Effect of discontinuation of anticonvulsants on late seizures

	No late seizures	Late seizures	*P*	OR	95% CI
Operation					
≤ 3 months	20	3		2	0.43–9.12
> 3 months	20	6	0.47	1	
Volume > 30 CC					
≤ 3 months	6	2		0.75	0.09–5.76
> 3 months	12	3	1	1	
Cortex					
≤ 3 months	22	4		3.05	0.81–11.3
> 3 months	18	10	0.12	1	

*OR* odds ratio, *CI* confidence interval

The results showed that discontinuation of VA within 3 months in patients with cortical involvement did not increase the risk of late seizures. Among 43 patients with cortical involvement and who used the prophylactic drug, in 11.5% (3/26) of patients seizures occurred after they discontinued prophylactic anticonvulsants, and in 58.8% (10/17) of patients seizures occurred during continued use of prophylactic anticonvulsants (Table 6).

**Table 6:** Characteristics of the 17 patients with late seizures

	Early seizures	Location	Anticonvulsant use	Seizures occurred	Anticonvulsant during seizure
1	Yes	Lobar	3 months	36 months	No
2	Yes	lobar	9 months	33 months	No
3	Yes	Lobar	3 years	11 months	Yes
4	Yes	Lobar	2 years	6 months	Yes
5	Yes	BG	3 years	20 months	Yes
6	No	BG	No	7 months	No
7	No	BG	1 month	2 years	No
8	No	BG	2 years	11 months	Yes
9	No	Lobar	No	13 months	No
10	No	Lobar	0.5 months	11 months	No
11	No	Lobar	4 months	3 months	Yes
12	No	Lobar	3 years	15 months	Yes
13	No	Lobar	2 years	21 months	Yes
14	No	Lobar	2 years	7 months	Yes
15	No	lobar	2 years	2 months	Yes
16	No	BG	2.6 years	7 months	Yes
17	No	Lobar	9 days	17 months	No

*BG* Basal ganglion

## Discussion

In the study, seizure attack was found in 13.6% (24/177) of patients with ICH, ES in 6.7% (12/177), and DS in 6.7% (12/177). ICH with cortical involvement is the only factor affecting early and late seizures. The incidence of seizure reported in our study is similar to those reported by studies by Qian et al. and Woo et al. [8, 14]. The ES rate was higher than that in the study by Zöllner et al. in Germany, which showed a 4% incidence rate of ES in patients with ICH. This was lower than that reported by the study by Herdt et al. [5], wherein 14% (71/522) of their patients had ESs. The difference is suspected to be attributed to most of the ES that occurred at stroke onset and within 24 h after stroke onset [5]. In the present study, we systemically interviewed the patients and their families about the occurrence of seizure, including seizure attack at stroke onset and before they arrived at the hospital. The study by Herdt et al. found that > 50% of ESs occur at stroke onset, whereas the study by Zöllner et al. only included seizures during inpatient treatment, which may have underestimated the patient seizure attack before they were brought to the hospital [19]. In our study, 41.7% (5/12) of patients with early seizure had late seizure within 36 months follow up. The recurrent seizure rate is higher than that reported by Kilpatric et al.; approximately 32% of their patients had late seizure [9]. The difference is suspected to be related to our study, which had a longer follow-up time.

Several studies have investigated early seizure and DS after ICH [5, 8, 10, 14]. In the present study, cortical involvement was found to be an independent factor for early, delayed, and late seizures. Previous studies have reported that with cortical involvement increases the risk of early and late seizures [5, 10, 26, 27]. ES after ICH is suspected to be related to hemorrhage with direct cortical irritation [5].

It is reasonable to consider that hemorrhage volume correlated with high NIHSS (National Institutes of Health Stroke Scale) score and high stroke severity. Nag et al. found that in patients with ICH NIHSS score ≥ 16, the mean hematoma is 29.03 CC. Hematoma volume ≥ 30 CC is associated with poor outcome [25, 28]. Our study found that hematoma volume ≥ 30 CC increased the risk of early seizures but not Delayed seizures. This is suspected to be related to hemorrhage with direct cortical irritation. Delayed and late seizures after ICH are suspected to be related to progressive neuronal and white matter damage due to small vessel disease, which amplify the epileptogenic process at the site of hemorrhage [10].

A previous study found young age (≤60 years) to be a predictor of seizure after ICH [14]. Our study also found that the patients who had DS were younger than those without DS, but this was not statistically significant.

Recently, most of the studies investigating prophylactic anticonvulsant in patients with ICH used levetiracetam for seizure prevention [19, 29, 30]. Jones et al. found that levetiracetam is as effective as phenytoin in the prevention of post-traumatic ES, but an EEG analysis showed that levetiracetam is associated with an increased seizure tendency [19]. Naidech et al. found that in comparison to levetiracetam, patients with ICH using phenytoin more frequently experienced in fever and poor outcomes [31]. In our study, our patients used VA for seizure prevention. We did not find an association between anticonvulsant and poor outcome but found that in patients with ICH with cortical involvement, discontinuation of prophylactic anticonvulsant within 3 months did not increase the risk of late seizure.

Although the guidelines for the management of ICH do not recommend that patients without seizures receive prophylactic anticonvulsant treatment [17], antiepileptic drug prophylaxis after ICH is common [32]. Previous studies found that prophylactic antiepileptic agent use was associated with a worse 3-month functional outcome [31, 33]. At present, there is no data available on the duration of prophylactic anticonvulsant in patients with ICH. In the present study, our results showed that the patients with ICH receiving prophylactic drugs for more than 3 months do not have a decreased risk of seizure compared to the patients who discontinued anticonvulsants within 3 months.

Our study has several limitations. First, the study is a retrospective study; we cannot measure VA serum level, and we did not carry out regular follow-up of electroencephalography (EEG), which may have affected the effects of VA. Whether regular follow-up EEG and adjustment of anticonvulsant dose by drug serum level can improve the preventive effects of anticonvulsants need further investigation. Second, the use of anticonvulsant was based on the physician’s decision, which may have introduced a bias of a higher stroke severity and larger hematoma volume in the patients receiving anticonvulsants and lower stroke severity and small hematoma volume in the patients not receiving anticonvulsant. In the present study, the stroke severity was significantly higher in the patients who received anticonvulsants than the patients who did not received anticonvulsants (*p* < 0.01) (Table S1). However, we compared the risk of seizures in patients with high seizure risk who used anticonvulsants and discontinued anticonvulsants before and after 3 months of ICH. Third, the study is a single-center study and included a small number of patients.

## Conclusion

Spontaneous ICH with cortical involvement may be a risk factor for early and late seizures. For preventing delayed and late seizures in patients with ICH, discontinuation of anticonvulsants in most patients within 3 months was adequate. Further prospective, randomized, double blind study to investigate the effect and timing of discontinuation of prophylactic anticonvulsant for seizure in patients with ICH may be necessary.

## Supplementary Information

**Additional file 1: Table S1**. Factors affecting the use of anticonvulsants

## Data Availability

All data used and/or analyzed in the manuscript are available from the corresponding author on reasonable request.

## Abbreviations

CI: Confidence interval
CT: Computed Tomography
DS: Delay seizure
ES: Early seizure
GCS: Glasgow Coma Scale
ICH: Intracerebral hemorrhage
OR: Odds ratio
SAH: Subarachnoid hemorrhage
VA: Valproic acid
