# Supplementary material for: Discontinuation of preventive antiepileptic drugs in patients with intracerebral hemorrhage
Source: BMC Neurol. 2021 Apr 7;21:150. doi: 10.1186/s12883-021-02177-w (PMC8025523; doi:10.1186/s12883-021-02177-w)
Supplement: Supplementary file 1 — Additional file 1: Table S1. Factors affecting the use of anticonvulsants [file 12883_2021_2177_MOESM1_ESM.rtf]

Table S1 Factors affecting the use of anticonvulsants
	No Anticonvulsants	Anticonvulsants	  p	
Sex				
 Men	    42	    77		
 Women	    22	    36	0.74	
Volumes				
 ≤30 CC	    63	    92		
 >30 CC	     1	    22	<0.01	
Operation				
 No	    61	    67		
 Yes	     3	    46	<0.01	
Cortex				
 No	    53	    70		
 Yes	    11	    43	0.004	
Late seizures				
 No	    62  	    98	0.03	
 Yes	     2	    15		
